# The connection between personal factors and burnout among medical workers during the COVID-19 pandemic

**DOI:** 10.1192/j.eurpsy.2024.1061

**Published:** 2024-08-27

**Authors:** E. V. Deshchenko, J. E. Koniukhovskaia, O. B. Stepanova, I. M. Shishkova, E. I. Pervichko, O. V. Mitina, E. R. Semenova

**Affiliations:** ^1^ Lomonosov Moscow State University; ^2^Higher School of Economics, Moscow; ^3^Ryazan State Medical University, Ryazan, Russian Federation

## Abstract

**Introduction:**

Work in the severe conditions of the pandemic has become a risk factor for the deterioration of the medical workers’ psychological state, which together can lead to professional burnout and, as a consequence, to professional mistakes (Pervichko, Konyukhovskaya, 2020).

**Objectives:**

The aim of the research was to study the connection between personal factors of medical workers and professional burnout during the COVID-19 pandemic.

**Methods:**

The degree of professional burnout was assessed using Maslach Burnout Inventory (MBI) (Maslach, 2000; Vodopianova, Starchenkova, 2008), HEXACO Personality Inventory (short version) was used to study personality traits (Ashton, Lee, 2007; Egorova, et al., 2019).

The study was conducted from May 2020 to October 2022. The sample consisted of 197 medical workers (32 men and 165 women), whose average age was 38.85±12.05.

**Results:**

Honesty as a personality trait is negatively significantly associated with emotional exhaustion (r=-0.268, p=0.000), depersonalization (r=-0.323, p=0.000) and positively associated with a smaller reduction in professionalism (r=0.290, p=0.000). Emotionality in medical workers is positively significantly associated with emotional exhaustion (r=0.358, p=0.000) and depersonalization (r=0.243, p=0.001) and with a greater reduction in professionalism (r=-0.380, p=0.000). Extroversion is negatively associated with emotional exhaustion (r=-0.478, p=0.000) and depersonalization (r=-0.376, p=0.000) and positively associated with a smaller reduction in professional achievements (r=0.566, p=0.000). Benevolence and conscientiousness reveal negative associations with depersonalization (r=-0.248, p=0.001; r=-0.180, p=0.012) and positive associations with a smaller reduction in professionalism (r=0.190, p=0.008; r=0.301, p=0.000).

**Conclusions:**

Thus, the state of emotional exhaustion during burnout is associated with greater emotionality, less honesty and extroversion. Whereas depersonalization and a greater negative assessment of one’s own professional competence and productivity is associated with less honesty, more emotionality, less extroversion, benevolence and consciousness.

Disclosure: Research is supported by the Russian Science Foundation, project No. 21-18-00624.

**Disclosure of Interest:**

None Declared

